# The Mary Newberry Trial

**Published:** 1893-07

**Authors:** D. R. Wallace

**Affiliations:** Waco, Texas


### TEXAS MEDICAL JOURNAL.

ESTABLISHED JULY, 1885.

PUBLISHED MONTHLY.—SUBSCRIPTION $2.00 A YEAR.

Vol. IX. AUSTIN, JULY, 1893. No. 1.

### Original Contributions.

For Texas Medical Journal.

### TRIAL OF MARY NEWBERRY.

BY D. R. WALLACE, M. D., LL. D., WACO, TEXAS.

"THE term insanity conveys quite different meanings to the community, to lawyers and physicians." * * *

"Cases involving the responsibility for crime are decided against science and the evidence, because of certain preconceived notions upon insanity which no amount of skilled opinion can controvert. Jurors, and less often judges, make up their mind what a sane man would do, under given conditions, and of what an insane man is capable, judging from the facts within their own experience; and in forming their decision it is the *act itself*, and not the man, diseased or otherwise, in connection with the act, that chiefly governs them. Often they are right,—not seldom wrong. Strange, illogical and inconsistent action is frequently attributed to the author of it being insane on the subject; whereas, he may be simply acting from strong impulse or emotion, and may be by no means insane. On the other hand, because a man knows right from wrong, and can ordinarily behave well, the very characteristic workings of his insane mind are often seized upon as unquestionable proof of sanity, even when they admit of no explanation to the skilled physician than that of insanity." * * * * * *

"With precisely the same degree of insanity and the same power to control their actions, two murderers may be sentenced,

—one to death for an act where the motive and method were those of the criminal, and the other to an insane asylum for killing a person under circumstances which are not explainable by sane reasons." * * * * * *

"To the lawyer insanity means only a condition of mind in reference to certain conduct. An insane man is simply *non compos mentis.* Insanity is irresponsibility. The lawyers' definition is narrower than that of the physician." * * *

"In the partly irresponsible condition of mind—often produced by grave hysteria, so-called nervous prostration, and the general mental and moral demoralization often seen in seduced and abandoned women, or after exhausting illness, or following apparent recovery from cerebral hemorrhages or embolism, blows upon the head, sunstroke, chronic alcoholism, syphilis, etc., there may be loss of self-control, or a distinct moral perversion, or a decided change of character without very evident mental impairment."

"In this connection the fact should be borne in mind that a very little mental disease can make bad people criminals, and may not take others beyond the bounds of propriety. A crimiinal may become insane and be still pretty much the same kind of criminal he was before; . . . . the perverse or criminal actions may be about equally explainable on the theory of insanity or wickedness." * * * * *

"The insane man often commits certain crimes precisely as an ordinary sane criminal would do the same thing." * * *

"The person alleged to be insane must, as a rule, be compared with himself at some previous time, and not with some ideal standard which does not exist. Indeed, if we could measure nicely, no two of us could be fairly held to precisely the same degree of accountability. . . . . A belief consistent with one person's whole life and character might indicate such a change in another as to be a mark of insanity." * * *

"It is important to the medical man to keep in mind that there may be a wide difference between medical insanity or mental disease, and legal insanity or irresponsibility. He does most wisely when he confines his testimony to an explanation of the changes caused by disease in the particular case, and to the effect of such changes upon the mind, leaving to the judge's charge and the jury's verdict the question of guilt and responsibility."—(*Chas. F. Folsom, Prof. of Psychology in Medical School of Harvard University.*)

"Compare a man with himself, his acts and thoughts now, with his acts and thoughts at some previous period, when his mind was in undoubted health; you will the better detect what is morbid, than if you set up a general comparison with the thoughts and acts of mankind."—(*Isaac Roy*, *Medical Jurisprudence.*)

---

"Reasoning madmen, whether the mind or morals be diseased. are always the most dangerous and difficult of the cases arising for adjudication." * * * * *

"Wm. Brown was executed at Maidstone, England, in 1812, for strangling a child he accidentally met one morning while walking in the country. On the trial he said he had never seen the child before; had no malice against it; could assign no reason for the dreadful act. . . . He told what he had done—requesting to be taken into custody. He bore an examplary character—never suspected of insanity."

"A young man in perfect health awoke suddenly one night in a fit of raving madness, ill-treated his wife—attempted to jump out of the window, and struck at whatever came in his way. Recovered within an hour and never had another attack." [Any number of similar cases can be given.—D. R. W.]

Such cases are confirmatory of Esquerol's opinion that "there exists a species of homicide madness in which no disorder of the intellect can be discovered." * * * *

"We believe that in the condition of the female system alluded to, acts are perpetrated as little according to the healthy, free and deliberate exercises of the individual, and for which they should be considered equally irresponsible; in which, previous to their commission, to all appearance, perfect mental and physical health existed; and subsequent to which a full consciousness and all the bitterness appertaining to the commission of the act are experienced." [Mary Newberry's case precisely.—D. R. W.]

"To those familiar with unsoundness of mind, it is matter of experience that by far the most dangerous class to be treated are patients who show the least evidence of their condition."

"It may appear, nay, it has been asserted, that there is a growing disposition to shield criminality beneath the plea of insanity. Several circumstances have conspired to favor this opinion. These circumstances rest in the marked increase in the number of the insane which late years have witnessed, and the juster estimate of the psychopathic relations of crime which their closer study of mental diseases enables physicians to form. The former is confirmed by the various asylums throughout the United Kingdom. The latter especially bears out the observation of Dr. Pritchard, who some years ago thus wrote: "We doubt not the time will come when the very names of many offenses against decorum, now punishable crimes, will be erased from the statute book; and when persons now liable to be sentenced to the pillory or the gallows will be treated as lunatics." Real practical benevolence finds in the application of medical truths additional incentives to commiseration. In their appreciation of the illegal acts of the insane, standing between the violated law and an outraged community, the psychopathic physician has on the most important trials, been compelled to listen to strictures on his evidence tantamount in many instances, to imputation against his integrity, from individuals whose position demanded that, if they did not promote, they should, at least, uphold those principles of justice, which the testimony of the physician has, in the exposition of insanity, so frequently preserved inviolate. . . . Medical science while rejecting fixed rules is dependent on just principles whose cultivation and application rest in the anxious and earnest study of each healthy as well as morbid vital process." * * * * * *

"Truth requires from justice that the insane be held, as regards the law, irresponsible. The object of both the lawyer and physician must be regarded for the purposes of justice and truth as being identical. At no period was it of more importance that the awakened intelligence of mankind which on all sides is active in pursuit of knowledge should know and feel that medical science despite the difficulty, if not obscurity, investing its practical elucidation is still in mental, as in bodily derangement, the instrument which should guide our proceedings, either for the restoration of individual health or the preservation of public safety. * * * * * * *

"We are constrained to believe that life and reputation have been at times sacrificed to the erring vengence of the law, rather than confided to the guarding care of the physician. . . . Studying in the great volume of nature written in *works* not *words*.

"J. W. Hume Williams,
of the Middle Temple, Barrister at Law, London."

These last excerpts are taken from a recent work, entitled "Unsoundness of Mind in its Legal and Medical Condititions." It will be noted that the writer is not a doctor, but a lawyer,—an eminent barrister of a city that may justly be styled the metropolis of the world.

These extracts from men eminent in law and medical science are placed as introductory to an account of one of the most extraordinary trials that ever took place in Texas, to show what ought to be the status of medical expert testimony in our law courts according to the highest authorities in medicine and law as well.

### THE BACKGROUND OF THE PICTURE.

Mrs. Mary Newberry was born in 1827. Was of humble, obscure parentage. Grew up amid the hardship of frontier life, without education, deprived of all the refining, elevating influences of civilized life. She can neither read nor write.

During the late war between the States,—living in Missouri, she suffered all the mischances and horrors incident to border warfare,—having witnessed numbers of those nearest and dearest to her murdered in cold blood.

Coming to Texas twelve years ago, she lived for first five years with her brother, Sam Barnard. From her brother's she went to live with his son, James Barnard, where she remained up to the time when, George Newberry, a younger son's wife dying, she removed to his place to take charge and care of his three children—the innocents murdered.

### COUNTY COURT SCENE—TRIAL UNDER CHARGE OF INSANITY.

Mrs. Newberry was committed to jail, charged with murder of her three grand-children. While awaiting proceedings in the district court, the question of her sanity was raised. A *writ de lunatico inquirendo* issued, and she was placed upon trial. At the instance of the county judge, the writer was employed to attend, examine the defendant, hear the testimony of witnesses, and give an opinion before the jury as a medical expert. Upon his arrival in Cleburne he had placed in his hands the testimony brought out in committing court trial—very voluminous—all of which was carefully read. He then visited the prison and passed nearly an hour with prisoner.

The case called and defendant placed upon trial, charged with insanity, the testimony disclosed about the same facts as those which had already been elicited in committing trial. As was to have been expected, her mental condition was known only to those who lived under same roof in daily intimacy. Exhibitions of insane conduct are not generally exposed to the view of strangers, or even neighbors, except it be in cases of wild mania, and even these are sometimes subdued or suppressed in such presence. The sort of insanity most dangerous, to a community and oftenest met in the criminal courts is not obvious to transient observation.

The testimony disclosed the following facts, to-wit:

That up to about twelve years ago, defendant was of a tender, gentle disposition and amiable deportment; useful in neighborhood in nursing the sick, of which she was fond, and for which she was noted wherever she had lived; and that she was seldom or never moved to anger.

That about the time she came to Texas, twelve years previous, then about fifty years old, the climacterium, she began to suffer with frequent attacks of a strange headache, with what she called a burning pain, which she could not describe, on the top of her head. During these paroxysms, she would keep her head bathed in camphor-spirits, and not only head, but all upper part of her body, wet with cold water. During one of these spells, she was severely cramped and violently convulsed, having what the doctor called a hysterical spasm. The whole time she was living at James Barnard's, she suffered in this way, the spells frequently recurrent and lasting from two to six days, during which she would sit with a sort of stony stare into vacancy. In this condition she was constantly picking at herself,—did not seem to know what she was doing; would pull scabs from her neck, put them in her mouth and chew them, and this not only before members of the family, but sometimes in the presence of visitors. Told of it, she would deny it, even while engaged in the act. Attempting to relate any occurrence, she would repeat over and again what was intended for same thing, but giving a different version,—seeming to have no mind and quite as little memory. She would change abruptly from one subject to another having no connection, and would frequently stop short in the midst of her talk, gazing about her idiotically.

That for days she could not be induced to go to the table, saying she was not hungry; but supposing herself unobserved, she would slip food from the cupboard. Asked why, she would deny doing it, even while eating.

That she would become angry with, and refuse to speak to members of the family, charging that they were talking about and trying to injure her. On one of these occasions, she made a bed on the floor in a little close room, of sweat-saturated saddle-blankets, in excessively warm weather in July, and remained there all day, resisting all efforts to induce her to leave her uncomfortable quarters. Upon another occasion, hearing a noise in her room, upon opening the door Mrs. Barnard saw she was entirely naked. She seemed greatly excited and frightened.

That these mental infirmities grew on her from year to year, becoming greatly aggravated the last year or two. She had, in early life, a fine mind and memory.

That like most grandmothers, she was very fond of these motherless grandchildren, and they of her, following her like her shadow everywhere she went. She lived with them on their father's place, situated in a thick wood half a mile from nearest house, which was James Barnard's, on whose place Geo. Newberry, the father of the murdered innocents, was farming.

That on the night of the killing, about 3 o'clock, the defendant came to James Barnard's in night clothes, bare of head and feet, collar and night gown bloody, a number of scratches and cuts on neck, seemingly in great distress of mind and of bodily exhaustion, and, though in warm July weather, shivering with cold, and said: "Two men came to Geo. Newberry's house, took me out, tied me to the gate, and, while one of them remained with and guarded me, choking and cutting me about the neck and throat, the other returned to the house and killed the children; on returning to the house after they had gone, I found the children dead."

That some days later, being very ill with one of her usual paroxysms, and thinking, as she stated, she was going to die, she called the family—James Barnard's—about her, and told them in the most solemn way that she was as innocent of the killing of the children as the angels in heaven.

That a day or two afterwards, she called James Barnard alone to her bedside, and told him "that she had been praying to the Lord to know whether or not she did kill the children; that something seemed to come down to her from heaven, telling her that she did it; that the children, too, had been with her in the night,—she was not mistaken, for she saw them, her eyes were wide open, she was not asleep,—they told her she did it, and must confess it, and not let the innocent suffer for the guilty, adding she could not tell why she did it, for she loved the children very dearly; the old devil must have made her do it." The next day she made a similar confession to the county attorney.

That since she confessed to the killing, she has expressed the greatest desire to expiate her crime by death, saying she was not fit to live.

These are the material,—there were a great number of other, circumstantial facts developed. There was a consensus of opinion among the medical men present, with an exception or two, and these held no definite opinion to the contrary,—an opinion based on the act itself and on the evidence as to her mental unsoundness for years prior,—*that the defendant, Mary Newberry, was insane.*

The venerable, learned and distinguished Dr. T. C. Osborn, was clearly of the opinion she was insane.

The accomplished physician and surgeon, Dr. T. J. Wayley, regarded the killing of grandchildren by grandmother as strong evidence of insanity, the annals of crime furnishing no parallel.

The writer gave it as his opinion that she was insane, and had been of more or less mental unsoundness for about twelve years.

She was declared by the jury to be insane. She was committed to the asylum at Terrell, Texas,—the North Texas Hospital for the Insane,—in February, 1892. Why this poor old unfortunate creature, the merest debris of a woman, a human wreck, should have been taken from this retreat, the only proper place for her, and dragged before the courts of the land, there may be those who can explain. But so it was.

The writer was attached and placed under a two hundred dollar bond to be at Cleburne to attend the December term of the district court of Johnson county, there to give evidence in the case of Mary Newberry vs. The State. (Were this the proper place he might stop to inquire: What right has the State of Texas to demand the special skill of one of her citizens—skill that is, presumably, the result of great labor and long study—without proper remuneration? The State, it may be mentioned, has taken months and months of this citizen's time without compensation. Query: Why not, with the same show of reason and justice, appropriate to the use of the State *all* his time, thus rendering him a pauper? Eminent lawyers say the State has no such right. The Supreme Court, it is known, has decided she has this right. What is a law-abiding citizen to do?)

To proceed. The writer was in the court room but a very short time before he realized—felt in the very atmosphere—that there had come about, in some way, quite a change in popular feeling, the prevailing one now being that this unfortunate woman must be prosecuted to the death; especially was this so among the ignorant and unthinking. It was very difficult to find twelve men *sufficiently ignorant* to serve on the jury. But after exhausting venires calling for hundreds—be it mentioned to the credit of Johnson county—the jury was at last empaneled.

The testimony as to the facts was substantially as in previous trials and *ut supra*.

The expert testimony was given in much greater detail. Previously, it was simply a question of mental soundness or unsoundness; now it is one of legal responsibility for crime,—not, to be sure, that the expert testimony is to decide that grave question, but it is expected of it that it will so explain the morbid condition, if such exists, as to help judge's charge and jury's verdict in the direction of right and justice. The testimony of the other physicians was substantially as on previous trial. Only Dr. T. C. Osborn's and the writer's was in detail and at length.

Dr. T. C. Osborn, called as an expert, testified as follows, viz: It is my opinion that Mrs. Mary Newberry was insane when she committed the homicide, and my reasons are like this:

1. It is in evidence that she, on many occasions, exhibited striking peculiarities of conduct, altogether different from her former character, for three or four years prior to the act of the homicide, known by the family of her son, and recognized by Dr. Towns, who was called to treat her on one occasion, and who testified that he warned her son of her insanity; and by Dr. Wallace, who in his testimony stated his belief that she had aberration of mind dating from her menopause, which occurred twelve years before the homicide; and in this opinion I concur with him, that in that interval she was suffering from progressive melancholia.

2. According to her own admissions, she "loved the three grandchildren better than anything in the world," and had no other motive for the deed than an uncontrollable impulse, which seemed to her as a dim dream; and it is well known that "many cases of insanity resemble waking dreams." I can therefore see no sane motive for committing the homicide, in any part of the testimony before the court; and when this is taken in connection with the bungling attempt at suicide, which she made immediately after the killing of the children, and her subsequent confession of the crime, there is but little doubt in my mind of her insanity at the time and for several years prior to the homicide.

3. It is not in testimony that she made any effort to escape, but was the first to inform her family of the killing; nor is it anywhere shown that she had accomplices; and these facts still further convince me of her insanity.

4. That she was laboring under an insane delusion, there can be but little doubt, as there is no evidence to show premeditation, precaution and concealment, other than a stout club found in the house, which is not shown to have been used in any way, and which I am at liberty to suppose was for use in her own protection, rather than as an instrument to kill the children with; but the deed was perpetrated with a butcher knife, and outside the house, while the children were asleep; and this also leaves me at liberty to suppose that she must have tenderly taken them, one at a time, outside the house, so as not to awaken them, having less in view the legal consequences of the act than the trouble it would give her to wash away the blood stains on the bed, and on the floor. This certainly looked very like a "waking dream," and in my opinion, was the act of an insane person, for even in slight degrees of insanity, in many cases there seems to be a loss of power controlling actions.

5. The three children being found side by side, dead, with their throats severed with a sharp instrument, the inference is that, beyond doubt, they were killed at the same time of the night; and this being established by the testimony of a number of witnesses, only one conclusion can be entertained, which is that it was the work of an insane person; for it is only such persons who kill several in such a manner at the same time, as sane people rarely kill more than one at a time, and it is always found in such cases that a sane motive actuated the deed.

These are my reasons for believing that Mrs. Newberry was insane at the time of the homicide, and that such instances of insanity never permanently recover from the mental aberration.

The writer testified as follows: Has been a physician for nearly forty years; has been a student of diseases of the mind and nervous system for nearly twenty-five years. Has had under his treatment and superintendence during this time about three thousand insane people. Has been in same house with insane people between ten and twelve years. He does not claim to be an expert in insanity. Has studied the subject, and lived many years with them, he may, therefore, claim, without immodesty, to know something of the insane—something about the workings of the insane mind. Clearly of the opinion that the defendant is, and has been for a number of years, of unsound mind. For this opinion, based, as it is, partly on personal examination, partly on the act for which she is arraigned, but mostly on the testimony of those who have lived in daily intercourse with her for years, it is not practicable to give his reasons in full; he will gladly answer any questions counsel may see proper to propound as to his reasons for this opinion, but to attempt to give them exhaustively would be nothing less than traversing the whole field of experience and professional studies.

There is no definition of insanity free from objections. The one having as few and as good for all practical purposes as any other is Hammond's, as follows: "A manifestation of disease of the brain, characterized by a general or partial derangement of one or more of the faculties of the mind, and in which, while consciousness is not abolished, mental freedom is weakened, perverted or destroyed." (Book not at hand, but this is the exact idea, and, it is believed, the exact words.)

The lawyer makes insanity the equivalent of irresponsibility. No so the doctor. There may be mental unsoundness or insaneness that does not destroy or interfere with the healthy exercise of the will to such degree as to destroy free agency. On the other hand, there may exist, along with apparent soundness of the intellect, or, at least, without very evident mental impairment, entire loss of self-control and moral perversion, so as to entirely change the character. That the defendant's mental trouble belonged to that class of unsoundness affecting the moral and emotional nature rather than the intellectual, goes without saying, —laboring, as she is, under what alienists term hysteria gravior —a disease of the mind at once the most hopeless and destructive to the more refined and elevated feelings, perverting the moral nature and changing the loveliest character into the most hateful and loathsome—the tender, amiable friend into the savage, vindictive fury. Such the general effect of this disease—the one with which the defendant is most unquestionably affected; it is not for the expert medical man to decide as to the question of legal responsibility. It is for the jury under the charge of the judge to determine to what extent this poor woman's moral nature was perverted and character changed, and therefore, to what extent, if at all, she is legally responsible for what would be criminal acts in a sane person.

In answer to question by counsel as to mental unsoundness and legal accountability the writer answered: "That the existence of mental unsoundness does not necessarily and in all cases imply legal irresponsibility, is generally accepted. Mental unsoundness is a variable quality. It may be complete, amounting to fatuity—entire mindlessness, gladdened by no hope, aroused into activity by no motive, stirred by no ambition;—it may be so slight as to escape the observation of the most intimate associate, and, if discoverable at all, so only to the scrutiny of the practiced eye of the psychologist. Between these extremes the degrees are as numerous, perhaps, as are the cases of mental unsoundness. To hold that all the manifold diversified cases are equally responsible before the law, were mere verbal trifling—coming little, if any, short of insisting that motive does not give moral complexion to individual acts. Would it not be nearer the mark to hold with the learned psychologist of Harvard University: "Indeed, could we measure nicely, no two of us could be fairly held to the same degree of accountability?"

The above contains a brief outline of the facts and statement of testimony of experts, so-called, before the court—fair and impartial to defendant and State.

J. W. Howe Williams, the learned London barrister, to whom we are so much indebted for the medico-legal doctrines that ought to underlie such investigations as those had in this noted case—quoting Baccaria's observation,—"The happiest of all nations is that in which the laws have not become a science," adds —"We are almost disposed to suspect that he had been studying the question of unsoundness of mind in its relation to responsibility for criminal acts; certainly to no subject is the remark more applicable; for, between the diversity of medical doctrines, antagomisms of legal opinions, uncertainty and difficulty which have been manifested in determining a majority of the cases which the records of criminal jurisprudence supply, one is led to the conclusion, that on this particular subject the lessons of experience have been strangely lost sight of and many doctrines perpetuated with inexplicable pertinacity in direct opposition to ordinary rules, which, in the every day affairs of life, regulate the affairs of men."

Could the distingushed barrister have been priviledged to be present at the trial of Mary Newberry, at the December term of the District Court of Johnson county, Texas, it is believed he would have witnessed a much more edifying exemplification of the language of the above quotation, than it has ever been his fortune to hear or read in his native country.

Was it because the laws of this country "have become a science" that common sense and reason seem to have been lost sight of in this trial and that the court-scene was turned into an arena not for administering of justice but for an exhibition of legal gymnastics.

The writer is not quite sure he comprehends what the learned jurisconsult meant by the words,—"laws have not become a science," but this much at least, it is believed, it is safe to infer he did mean:—That nation is the happiest, or best governed, affairs best regulated, whose laws have not crystallized into shapes hardened into forms and stiffened into methods of such inflexible rigidity as renders them unsuitable to and incapable of application in the complicated affairs and conduct of organized society. Forms must be observed—justice can beg:—is this the meaning? Few persons could have witnessed the trial of Mary Newberry free from such feeliug. It seemed to the writer that the methods employed were not used for arriving at the facts, but for the suppression of truth. Was it from having witnessed many such court scenes that the most eminent jurist, perhaps that Texas ever had, and for many years supreme judge, after long experience on the district bench, said to the writer that he believed that justice would be more nearly done between man and man, if there was not a law book in the State. It cannot be certainly, that an officer of the court is really more anxious for successful prosecution than that a poor old unfortunate woman should have justice done by her. If she committed the deed, the act being without cause or motive, proves her insane. If she confessed to its commission when not guilty, this showed her to be insane. Just as a century or two ago thousands of crazy old women were burned as witches upon their confession of being guilty of witchcraft. The previous history of the defendant too as sworn to by those living in the same house with her showed beyond a reasonable doubt she was insane when the act was committed, and had been for years. Yet when an aged medical man, no small portion of whose professional life of forty years nearly, has been spent under the same roof with insane people gives it as his opinion, based upon all this evidence, reinforced by a personal examination, that she was when the act was committed, and still is insane, the prosecuting attorney, fearing, it would seem, the effect of this evidence with the jury, asked with a very grave look and significant tone of voice, "Doctor, what is your religion?" Did he think because this witness belonged to no church and professed no creed, that he was less capable of telling the truth than if he repeated night and morning| on his knees the whole Athernasian creed and swore by the thirty-nine article of faith? Or did he wish to prejudice an ignorant, superstitious jury? It is true, this witness is known, where he is known at all, as belonging to no creed, in fact, as not setting himself up as knowing any great deal about certain matters and events claimed in the name of religion to have happened some thousands of years ago; yet, it is believed he has led a blameless life—a gentleman whose honor and integrity have never been questioned. The writer is glad to be able to state that the good lawyer did not ask the witness if he had driven off any sheep or cows that did not belong to him, stolen any horses, or robbed any hen-roosts. Perhaps he knew these questions would not serve his purpose so well with the jury. To be sure the question was objected to and the court instructed the witness not to answer. It was not expected, of course, the question would be answered. The purpose for which it was put was accomplished—*it got in its work.*

The writer would be understood. The prosecuting attorney was a pleasant amiable gentleman, and for his age, fine lawyer, and outside the courthouse treated witness with marked courtesy. It was not the *man* but the lawyer that is complained of, not the lawyer so much as the *methods* of the law.

That there should be "diversity of medical doctrines and antagonisms of legal opinions," in causes involving the question of responsibility for acts committed by persons of doubtful mental soundness is no more to be wondered at than that intelligent persons should honestly differ in regard to any other matter of inquiry in which the facts are obscure and conflicting, or of difficult or doubtful interpretation. This difficulty, it is believed, does not constitute the chief trouble in such causes in the law courts.

There would be less "diversity of medical doctrines" were only competent medical men called to expound them. That ignorant doctors should be called to the witness stand as experts, to enlighten judge and jury, in regard to questions involving matters of which they are profoundly ignorant, simply because they can be induced from their relations to the parties litigant to give certain opinions,—this it is mainly that diversifies medical doctrines, brings reproach upon medical expert testimony, and has caused eminent legal authorities to hold it in low esteem, if not of absolute contempt,—as was expressed by Judge Davis, of the supreme court of Maine, in delivering the opinion in Neal's case. He said: "If there is any kind of testimony that is not only of no value, but worse than that, it is, in my judgment, that of medical experts. They may be able to state the diagnosis of a case more learnedly, but upon the question, whether it had at a given time reached such a stage that the subject of it was incapable of making a contract or irresponsible for his act, the opinion of his neighbors, if men of good common sense, would be worth more than all the medical experts in the country." Similar expressions of the estimate in which eminent legal authorities hold such testimony might be quoted ad infinitum. But the opinion is ventured, they were not based upon medical expert testimony in any proper sense of the word. On the contrary, facts bear out the statement that there is fully as great consensus of opinion among real medical experts as there is among other experts in questions of difficult determination. "The term medical expert may be defined as meaning, one who, by reason of knowledge, skill or experience in medicine is considered by law to be a proper exponent of questions relating thereto." Take the medical expert testimony one hears in the courts of Texas upon the question of responsibility for acts committed by persons of doubtful soundness of mind, and no well informed right thinking person, doctor or not, but would quite agree with Judge Davis in his opinion of it. To call such expressions of opinion on the part of persons with no knowledge of the subject and no other qualification to give testimony medical expert evidence, is to use words without meaning.

In a paper entitled "Medical Expert Testimony, or the Doctor in Court," published some years ago, the writer said:—"Is there any role in which the medical man cuts so shabby a figure, appears to such disadvantage, as when he comes forward as a medical expert in a case in regard to which he has no special information? It is a scene provocative of mirth and ridicule for lawyers and spectators, but, in all conscience, humiliating to the last degree to the true medical man who cares for the honor of his profession. Who that is worthy of the title of M. D. can behold, without a blush, an ignorant creature, as innocent of a knowledge of scientific medicine as of the flora and fauna of the north pole, setting himself—a spectacle for men and Gods—as a medical expert in a cause involving principles of which he has not one intelligent idea?

"No medical man, whatever may be his eminence in some one special department of his profession, should allow himself to appear as an expert in a law court in some other in which he knows himself deficient in the necessary information. Dr. A. is an expert surgeon, follows it, as a matter of course, he is an analytical chemist? Dr. B. is a skilful practitioner of general medicine but it does not follow that his opinion is of any special worth in a case of malpractice in surgery. Dr. C. has a national reputation as a gynecologist; it is hardly probable that he is an expert as a psychologist.

---

It only remains to state that this *remarkable* jury in this *remarkable* case brought in the *remarkable* verdict: "Guilty of murder in the first degree and assessed the punishment at confinement in the penitentiary for life."

It is hardly necessary to state—certainly not to those who are acquainted with the eminent gentlemen and learned jurists constituting our appellate bench—that they very promptly handed down an opinion reversing the verdict.

It it believed Judge Hall should at once order Mary Newberry back into the custody of the Insane Hospital at Terrell, the Superintendent of which is bound to admit her. He has no discretion in the premises. Such, at least, was the opinion, some years ago, of one of the most eminent attorney-generals that ever held office in Texas.

